# Developing chicken cardiac muscle mitochondria are resistant to variations in incubation oxygen levels

**DOI:** 10.1016/j.crphys.2022.03.001

**Published:** 2022-03-17

**Authors:** Vanessa J. Starr, Edward M. Dzialowski

**Affiliations:** Developmental Integrative Biology, Department of Biological Sciences, 1155 Union Circle #305220, University of North Texas, Denton, TX, 76203, USA

**Keywords:** Cardiac mitochondria, Chicken, Hypoxia, Hyperoxia, Reactive oxygen species, AA, Antimycin A, ADP, adenosine diphosphate, dph, days post hatching, COX, cytochrome oxidase, EP, external pipping, GMP, glutamate, malate, and pyruvate, IP, internal pipping, LEAK, mitochondrial leak respiration, OXPHOS, mitochondrial oxidative phosphorylation, OMY, oligomycin, ROS, reactive oxygen species, ROT, rotenone, S, succinate, TMPD, N,N,N^’^,N^’^-tetramethyl-p-phenylenediamine

## Abstract

**Background:**

Chronic exposure to hypoxia during vertebrate development can produce abnormal cardiovascular morphology and function. The aim of this study was to examine cardiac mitochondria function in an avian model, the chicken, in response to embryonic development under hypoxic (15% O_2_), normoxic (21% O_2_), or hyperoxic (40% O_2_) incubation conditions.

**Methods:**

Chicken embryos were incubated in hypoxia, normoxia, or hyperoxia beginning on day 5 of incubation through hatching. Cardiac mitochondria oxygen flux and reactive oxygen species production were measured in permeabilized cardiac fibers from externally pipped and 1-day post hatchlings.

**Results:**

Altering oxygen during development had a large effect on body and heart masses of externally pipped embryos and 1-day old hatchlings. Hypoxic animals had smaller body masses and absolute heart masses, but proportionally similar sized hearts compared to normoxic animals during external pipping. Hyperoxic animals were larger with larger hearts than normoxic animals during external pipping. Mitochondrial oxygen flux in permeabilized cardiac muscle fibers revealed limited effects of developing under altered oxygen conditions, with only oxygen flux through cytochrome oxidase being lower in hypoxic hearts compared with hyperoxic hearts. Oxygen flux in leak and oxidative phosphorylation states were not affected by developmental oxygen levels. Mitochondrial reactive oxygen species production under leak and oxidative phosphorylation states studied did not differ between any developmental oxygen treatment.

**Conclusions:**

These results suggest that cardiac mitochondria function of the developing chicken is not altered by developing *in ovo* under different oxygen levels.

## Highlights

• Chicken heart mass is influenced by oxygen availability during development.
• Cardiac mitochondria respiration was unchanged by developing under hypoxic or hyperoxic oxygen stress.
• Cardiac mitochondria ROS production was not altered by developmental oxygen stress.

## 1 Introduction

Developing under intrauterine growth restriction or low oxygen conditions produces deleterious effects on the cardiovascular system of developing amniotes (Giussani et al., 2012; Giussani and Davidge, 2013; Louey and Thornburg, 2005). Exposure to chronic hypoxia during fetal development can produce negative cardiovascular effects at the fetal and neonatal stages (Thompson et al., 2018, 2020) and later into the juvenile and adult stages (Giussani and Davidge, 2013; Hellgren et al., 2021). Hypoxia during prenatal development remodels cardiac tissue in fetal and neonatal rats (Tong et al., 2011). Chicken embryos developing under hypoxic 15% O_2_ conditions exhibit gross cardiomyopathies, such as increased left ventricular dilation, reduced ventricular wall mass, and increased apoptosis that last into adulthood (Tintu et al., 2009). Exposure to 15% O_2_ during development reduces left ventricle function compared to normoxic counterparts in chicken embryos (Jonker et al., 2015).

Even though gross morphology and some physiological function is altered in response to developing under chronic hypoxia, the fetal cardiac myocytes of mammals and birds are adapted to function under what would be considered internal hypoxic conditions when compared with adult oxygen levels (Comline and Silver, 1970; Copeland and Dzialowski, 2009; Kawashiro and Scheid, 1975; Tazawa et al., 1983), relying upon glycolysis (Breckenridge et al., 2013; Lopaschuk et al., 1991). There is evidence that developing in hypoxic conditions can have either no effect or a detrimental effect on cardiac mitochondria function in mammalian species (Al-Hasan et al., 2013, 2014; Galli et al., 2016; Thompson et al., 2018, 2020). Little is known about how cardiac mitochondria from developing birds responds to development under altered oxygen conditions. Cardiac mitochondria from another archosaur, the American alligator, shows limited effects of incubating under hypoxic conditions (Galli et al., 2016). Developing under hypoxia in mice and snapping turtles was found to alter mitochondrial function in the adult, but was not studied in the embryo or neonate (Galli et al., 2021; Hellgren et al., 2021).

In the present study, we examined the effect of incubating chicken embryos under 15% O_2_ hypoxia on the development of cardiomyocyte mitochondrial function. Because hyperoxic exposure can also alter mitochondria function (Das, 2013; Ratner et al., 2009; Turrens et al., 1982), we also examined the mitochondria response of cardiomyocytes to 40% O_2_ hyperoxia exposure during development. Our findings suggest that chicken cardiac mitochondria are resistant to chronic altered oxygen exposure during *in ovo* development.

## 2 Materials and methods

### 2.1 Animal care

Fertilized White Leghorn chicken eggs were obtained from Red Bluff Farm (Iowa Park, TX, USA) and the Department of Poultry Science at Texas A&M University (College Station, TX). Eggs were weighed on day 0 of incubation and incubated at 20.95% oxygen in a circulated air incubator (Model 1502, G.Q.F. Manufacturing Company, Inc., Savannah, GA, USA) until day 5 of development (average temperature 37.4 °C, 59% relative humidity (RH%)). On incubation day 5, viable eggs were randomly placed in circulated air Hova-Bator incubators (Model 2365, G.Q.F. Manufacturing Company, Inc., Savannah, GA, USA) maintained at either 20.95% O_2_ normoxia, hypoxia of 15% O_2_, or hyperoxia of 40% O_2_ for the remainder of incubation. These levels of oxygen have been shown to produce arterialized pO_2_ levels of 48 mmHg in 15% oxygen, 63.9 mHg in normoxia, and 85.7 mmHg in 30% oxygen on day 16 of incubation (Copeland and Dzialowski, 2009). Oxygen levels for all Hova-bators were maintained using a ROXY-4 channel gas regulator (Sable Systems International, North Las Vegas, NV, USA), and monitored using Lab Chart 7 software (version 7.3.7, ADInstruments, Colorado Springs, CO, USA) with a PowerLab 8SP (ADInstruments, Colorado Springs, CO, USA). The O_2_ electrodes (Max 250S Oxygen Sensor, MAXTEC, Salt Lake City, UT, USA) were calibrated weekly at 20.95% oxygen. Eggs were automatically turned every 4 h. All experiential procedures were approved by the UNT IACUC.

During developmental days 18 through 20, eggs were checked daily for viability, internal pipping (IP) of the air cell, and external pipping (EP) of the eggshell. The EP stage embryos were utilized once external pipping was observed between days 20 and 21. To obtain the 1-day old hatchlings, EP stage eggs were moved to a clear Lyon hatching incubator (Lyon Technologies, Inc., Chula Vista, CA, USA) maintained at an average temperature of 37.0 °C, an average RH% of 65%, and 21% O_2_ with time-lapsed photographs taken at 30-min intervals to determine the time of hatching within a 30 min window. Hatchlings were maintained in a Hatchrite incubator (maintained at 35 °C with a daily 12-h light and 12-h dark cycle) with ad libitum access to food and water until use at 1 day post hatch (dph) considered to be between 24 and 48 h after hatching.

### 2.2 Permeabilized cardiac muscle fiber oxygen and ROS flux

The modified protocol for permeabilization of cardiac muscle fibers and subsequent measurements of mitochondrial respiration and hydrogen peroxide (ROS) production was adapted from (Sirsat et al., 2016) and Hickey et al. (2012). Chemicals were obtained from Sigma-Aldrich (Millipore Sigma, St. Louis, MO, USA), unless otherwise noted.

White Leghorn chicken EP embryos and 1 dph hatchlings were euthanized by inhalation overdose of isofluorane followed by decapitation, after which the masses of the yolk sac, heart ventricles, and yolk-free body masses for EP embryos and 1 dph hatchlings were measured. The atria, connective tissue, and blood vessels were removed from the heart ventricles and placed in a Petri dish containing ice-cold BIOPS solution (mmol l^−1^: 50 4-morpholineethanesulfonic acid potassium salt, 20 taurine, 0.5 dithiothreitol, 6.56 magnesium chloride hexahydrate [MgCl_2_ x 6H_2_O], 5.77 adenosine 5^’^-triphosphate disodium salt hydrate [Na_2_ATP], 15 phosphocreatine disodium salt hydrate [Na_2_-phosphocreatine], 20 imidazole, 2.77 CaK_2_EGTA, and 7.23 K_2_EGTA; final pH 7.1). The left ventricle cardiac muscle fibers were teased apart under a Leica EZ4 stereo microscope (Leica Microsystems, Inc., Buffalo Grove, IL). Teased cardiac muscle fibers were placed in 2 ml of BIOPS containing saponin (50 μg ml^−1^) solution for 20 min to permeabilize the fibers. This was followed by three successive 10-min washes in 2 ml of MiR05 (mmol l^−1^: 20 4-(2-hydroxyethyl)piperazine-1-ethanesulfonic acid (HEPES), 20 taurine, 110 D-sucrose, 60 potassium lactobionic acid, 0.5 EGTA, 3 MgCl_2_ x 6 H_2_O, 10 potassium dihydrogen phosphate [KH_2_PO_4_], and 1 g l^−1^ bovine serum albumin (BSA) without fatty acids) at 4 °C with continuous rocking (Bio-Rad UltraRocker, Bio-RAD Laboratories, Hercules, CA, USA). Three to 5 mg of permeabilized left ventricle cardiac muscle fibers from each subject were blotted dry on Kimwipes and then weighed using a dual-range semi-micro balance (Model XA105, Mettler-Toledo, LLC., Columbus, OH, USA). The sample was added to a chamber of a high-resolution respirometer Oroboros oxygraph-2K (Oroboros Instruments, Innsbruck, Austria) containing 2 ml of MiR05 respiration medium. Mitochondrial respiration and reactive oxygen species (ROS) production at 38 °C were measured simultaneously for each sample. To ensure that oxygen was not limiting, chamber oxygen levels were maintained between 250 and 400 nmol ml^−1^ by adding supplemental oxygen at the beginning of the run and when oxygen levels reached 250 nmol ml^−1^ (Pesta and Gnaiger, 2012).

**Fig. 1:**
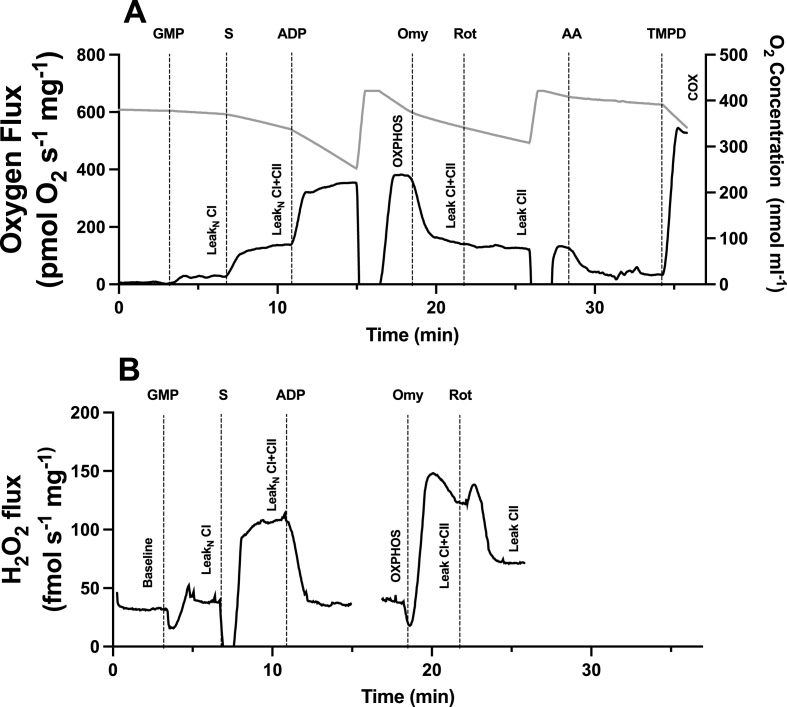
Representative traces for substrate inhibitor protocol used to measure (A) mitochondrial respiration oxygen flux and (B) mitochondrial total reactive oxygen species (H_2_O_2_) flux in permeabilized cardiac ventricle muscle fibers of externally pipped chicken embryos and day-old chicken hatchlings. The substrate inhibitor titration protocol consisted of the addition of glutamate, malate, and pyruvate (GMP) supporting Complex I flux in the leak state without ADP (LEAK_N-CI_), followed by addition of succinate (S) for Complexes I and II flux in the leak state without ADP (LEAK_N-CI + CII_). ADP (D) was added to support oxidative phosphorylation (OXPHOS) through Complexes I and II. Oligomycin (OMY) and rotenone (Rot) were given for leak states through Complexes I and II (LEAK_CI + CII_) and Complex II (LEAK_CII_). Antimycin was provided followed by N,N,N,N′-tetramethyl-p-phenyldiamine and ascorbate (TMPD) to determine maximal oxygen flux through cytochrome oxidase (COX). The chamber was reoxygenated at two points during the run to maintain chamber oxygen above 250 nmol ml^−1^.

Mitochondrial oxygen flux was measured using a multiple substrate-inhibitor titration (SUIT) protocol (Fig. 1A). Mitochondrial leak respiration (LEAK_N_) through complex I without ADP (LEAK_N-CI_) was stimulated by adding the substrates glutamate (10 mmol), malate (2 mmol), and pyruvate (5 mmol). LEAK through complexes I and II without ADP (LEAK_N-CI + CII_) was then stimulated by addition of succinate (10 mmol). Oxidative phosphorylation through complexes I and II (OXPHOS_CI + CII_) was stimulated by adding adenosine diphosphate (ADP, 2.5 mmol). LEAK_CI + CII_ was then stimulated by the addition of the ATPase complex V inhibitor oligomycin (OMY, 2.5 μmol). LEAK_CII_ was measured following addition of the complex I inhibitor rotenone (ROT, 0.5 μmol). Residual oxygen consumption was measured after the addition of Antimycin A (AA Residual, 2.5 μmol). Finally, maximum cytochrome oxidase flux (COX) was stimulated with addition of N,N,N^’^,N^’^-tetramethyl-p-phenylenediamine (TMPD, 0.5 mmol) and ascorbate (2 mmol).

To detect ROS production Amplex UltraRed (10 μmol), horseradish peroxidase (1 unit ml^−1^), and superoxide dismutase (5 units ml^−1^) were added to each chamber at the start of each run (Fig. 1B). Reactive oxygen species accumulation was measured simultaneously with oxygen flux using purpose-built fluorospectrometers as in (Hickey et al., 2012). The chamber was illuminated with a light emitting diodes to provide excitation at 520 nm. Emitted light was then detected using a photodiode placed adjacent to the excitation diode covered with a 590 nm low pass filter. The signal was amplified by the oxygraphy and recorded in DATLAB. To calibrate the fluorospectrometer, 0.1 μmol H_2_O_2_ was added to each chamber prior to addition of the SUIT substrates. All ROS fluxes were subtracted from a baseline ROS flux measured without any mitochondria substrates.

### 2.3 Statistical analyses

We analyzed the variable responses using ANOVAs and Cohen's d with 95% confidence intervals, a measure of effect size. We used effect size measures to assess the magnitude of the effect of altering oxygen levels on cardiac growth and function (Nakagawa and Cuthill, 2007; Sneddon et al., 2017). For the Cohen's d effect sizes we classify the following ranges for small (0.2 < d < 0.49), moderate (0.5 < d < 0.79), large (0.8 < d < 1.19), or very large (d > 1.2) effects (Nakagawa and Cuthill, 2007). Body mass and heart mass was analyzed by two-way ANOVA with age and oxygen level as factors. Heart mass was also analyzed by two-way ANCOVA with yolk-free body mass as a covariate to remove the effect of body mass. The omnibus tests were followed by pairwise multiple comparison procedure by Sidak *post hoc* test. Morphometric data is presented as either the absoulte means or ANCOVA estimated marginal means ± 95% confidence intervals. For heart mass, Cohen's d ± 95% CI was determined for the absolute means and the ANCOVA estimated marginal means. We considered Cohen's d effect sizes of importance when the ±95% CI did not encompass 0. We analyzed mitochondrial oxygen flux and ROS production using Cohen's d effect sizes (Nakagawa and Cuthill, 2007). Sample sizes varied for each variable measured across treatments and ages and are provided in the figure legends in which those data are presented. The level of significance was set at *P* < 0.05. All analyses were run in Jamovi running R version 4.0.2.

## 3 Results

### 3.1 Morphology

**Fig. 2:**
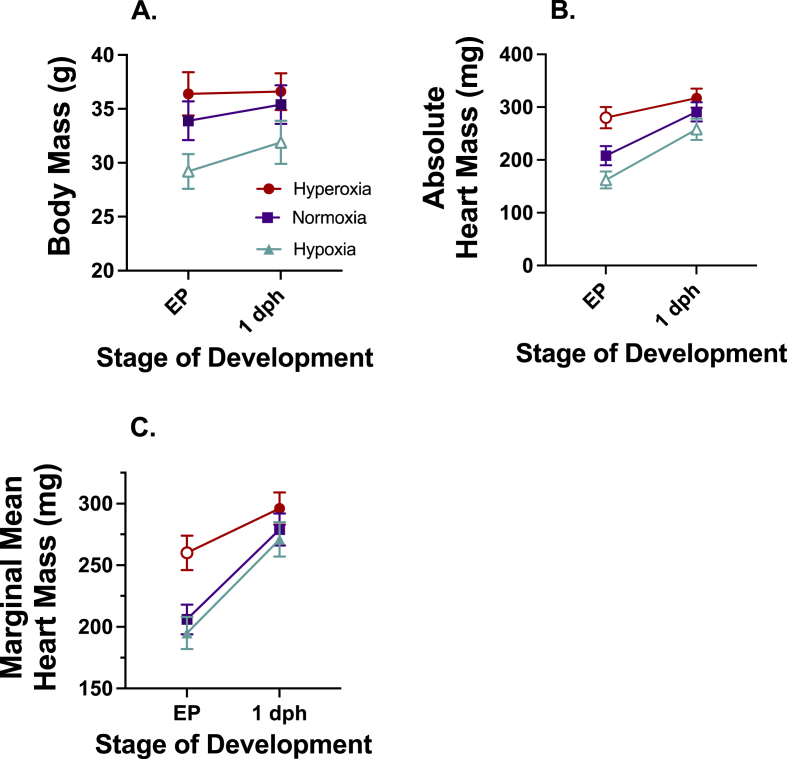
Yolk-free body mass, absolute heart mass, and body mass corrected estimated marginal mean heart mass of EP and 1dph hatchlings incubated in hypoxia (15% O_2_; EP n = 24; 1 dph n = 16), normoxia (21% O_2_; EP n = 19; 1 dph n = 19), or hyperoxia (40% O_2_; EP n = 15; 1 dph n = 21). Incubation in hypoxia or hyperoxia had a large effect on (A) body mass, (B) absolute heart mass, and (C) mass corrected heart mass development. Heart masses presented as either (B) absolute or (C) estimated marginal means ± 95% confidence intervals and body mass as absolute means ± 95% confidence intervals. Open symbols indicate a very large effect of hypoxia or hyperoxia compared with the normoxic values determined by Cohen's d.

Development under altered oxygen levels had a large effect on both body mass (Fig. 2A; df = 2, 108; F = 22.04; p < 0.001), absolute heart mass (Fig. 2B; df = 2, 108; F = 44.53; p < 0.001), and ANCOVA marginal mean heart mass (Fig. 2C; df = 2, 107; F = 20.29; p < 0.001). The effect of hypoxic incubation on yolk free body mass of externally pipped embryos and 1 dph hatchlings were very large when compared with control (EP d = −1.18 [−1.81, −0.55]; 1dph d = −0.89 [−1.56, −0.20]) and hyperoxic (EP d = −1.82 [−2.52, −1.13]; 1dph d = −1.19 [−1.87, −0.52]) incubated animals (Fig. 2A). When looking at absolute heart mass at the EP stage (Fig. 2B), developing under hypoxia (d = −1.14, [−1.76, −0.51]) and hyperoxia (d = 1.80, [1.08, 2.53]) had very large effects on absolute heart mass when compared with the normoxic EP animals. At 1 dph, hypoxia had a large effect on absolute heart mass (d = −0.82, [−1.50, −0.14]) when compared with the normoxic hearts. Hyperoxic hearts were only moderately larger than the normoxic hearts on 1 dph (d = 0.65 [0.01, 1.28]). Hyperoxic incubation had a very large effect on ANCOVA body mass corrected heart mass when compared with both normoxic and hypoxic incubated animals (Fig. 2B). At the EP stage, both hypoxic (d = −2.33 [−3.14, −1.53]) and normoxic (d = −1.92, [−2.66, −1.78]) incubated embryos had similar sized, smaller hearts than hyperoxic incubated embryos. In 1-day old hatchlings, the there was little effect of oxygen on the body mass corrected heart masses.

### 3.2 Mitochondria physiology

**Fig. 3:**
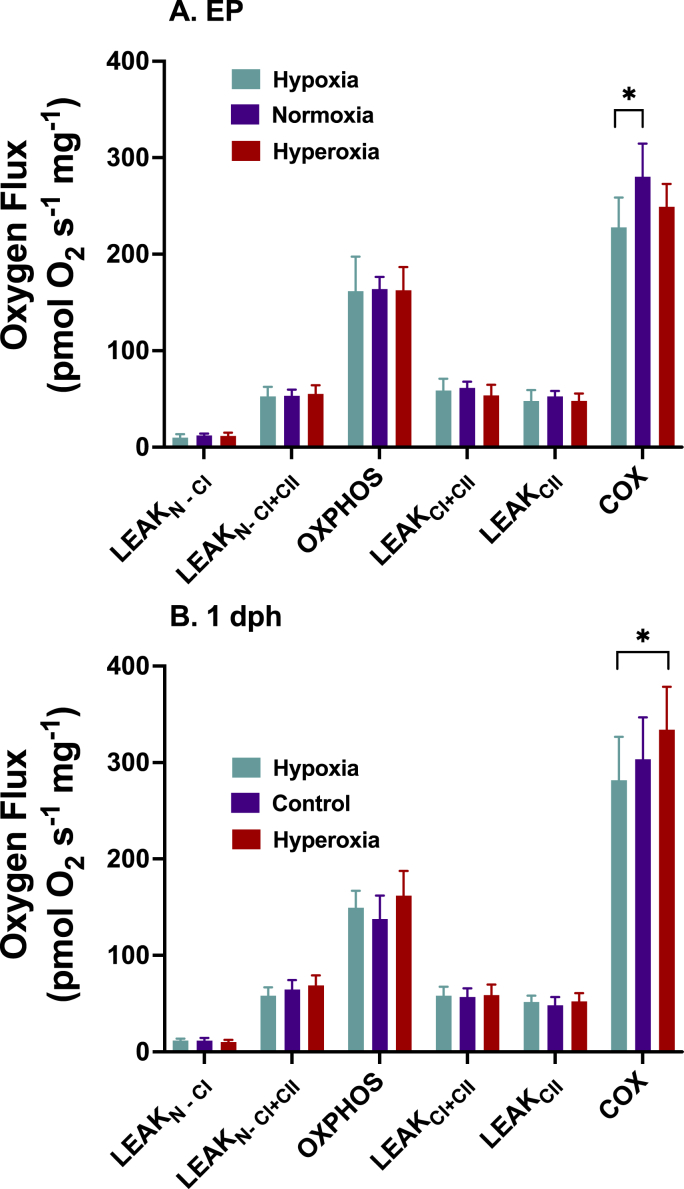
Mitochondria respiration of permeabilized cardiac ventricle fibers from A) EP and (B) 1 day old chicken hatchlings incubated in 15% O_2_, 21% O_2_, or 40% O_2_. Mitochondrial O_2_ flux only differed between incubation treatments at cytochrome *c* oxidase. Sample sizes are EP-hypoxia n = 21; EP-normoxia n = 22; EP-hyperoxia n = 14; 1dph-hypoxia n = 15; 1dph-normoxia n = 19; 1dph-hyperoxia n = 21. Data presented as mean ± 95% confidence intervals. * indicates a very large effect between the two treatments determined by Cohen's d. Refer to Fig. 1 legend for description of the x axis abbreviations.

Development under altered oxygen levels has little effect on the cardiac ventricle mitochondrial oxygen flux (Fig. 3). The only effect of oxygen level was seen in the cytochrome oxidase, Complex IV, oxygen flux of the externally pipped embryos (df = 2, 51; F = 4.81; p = 0.012). Cytochrome oxidase oxygen flux of hypoxic externally pipped embryo mitochondria was lower than normoxic mitochondria (d = −0.98 [−0.43, −1.53]). In 1 dph cardiomyocyte mitochondria, there was a large effect of oxygen status on cytochrome oxidase activity when comparing hypoxic to hyperoxic animals (d = −0.81 [−1.40, −0.22]). There was no effect of oxygen incubation levels on any of the other states of mitochondrial oxygen flux (all p > 0.05; d < 0.65).

**Fig. 4:**
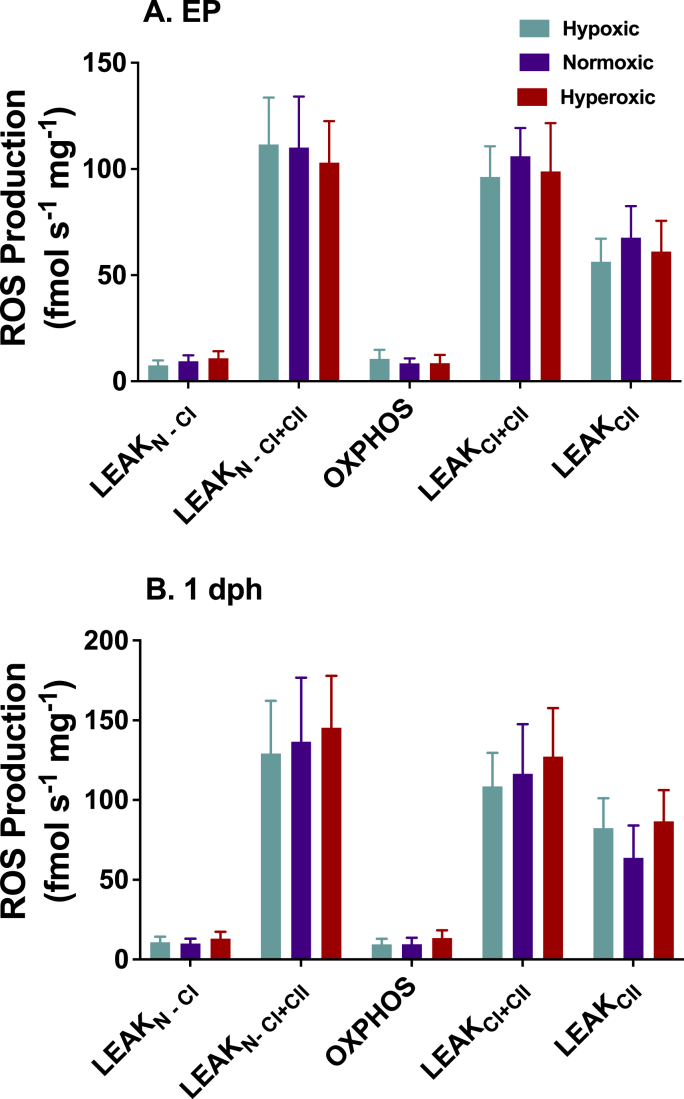
Mitochondrial reactive oxygen species (ROS) flux in permeabilized cardiac ventricle fibers from A) externally pipped and (B) 1 day old chicken hatchlings incubated in 15% O_2_, 21% O_2_, or 40% O_2_. There was no effect of incubation O_2_ level on the production of ROS at either age. Production of H_2_O_2_ was greatest in the leak states in the presence of a complex II substrate. Refer to Fig. 1 legend for description of the x axis abbreviations and associated substrates and inhibitors. Sample sizes are EP-hypoxia n = 19; EP-normoxia n = 22; EP-hyperoxia n = 14; 1dph-hypoxia n = 17; 1dph-normoxia n = 16; 1dph-hyperoxia n = 16. presented as mean ± 95% confidence intervals.

Reactive oxygen species production was greatest in the LEAK states involving complex II (Fig. 4). Development under altered oxygen levels had no effect on the production of reactive oxygen species under LEAK or OXPHOS respiration states compared with normoxia (all p > 0.05; d < 0.65).

## 4 Discussion

### 4.1 Effects of developmental hypoxia and hyperoxia on morphology

Our results found that altering the level of developmental oxygen influenced gross morphology of the developing heart but had only limited influence on cardiac mitochondrial physiology of embryonic and neonatal chickens. Hypoxic incubation slowed growth, with hypoxic externally pipped embryos and hatchlings having smaller masses than normoxic and hyperoxic animals. After removing the influence of body mass, hyperoxic embryos had larger hearts than either normoxic or hypoxic animals, similar to that found in another study when incubating at an even higher level of 60% O_2_ for the last 5 days of incubation (Asson-Batres et al., 1989). Hypoxic and normoxic hearts were of similar size after removing the effect of body mass. The mass responses of heart mass to hypoxic incubation are variable in the chicken. Some studies have found larger hearts in hypoxic incubated near-term chicken embryos and hatchlings (Dzialowski et al., 2002; Jonker et al., 2015; Rouwet et al., 2002) while others have found no differences (Chan and Burggren, 2005). Differences in hypoxic and normoxic heart masses have been observed in another archosaur, the American alligator, where hearts were larger in hypoxic incubated embryos compared with normoxic embryos when controlling for body mass (Galli et al., 2016). Conflicting findings on the effects of hypoxia and intrauterine growth restriction have been seen in mammalian studies as well. In rats, studies have found both an effect of hypoxic exposures during *in utero* development on relative heart mass (Bae et al., 2003) and no effect on heart mass (Giussani et al., 2012). In fetal sheep hearts under a hypoxic growth-restriction model, the heart masses did not differ, but there were changes in the underlying cellular structure of the cardiac muscle cells (Morrison et al., 2007). It remains to be seen the extent of the underlying cellular changes brought about by hypoxic and hyperoxic incubation in the developing chicken heart.

### 4.2 Mitochondrial respiratory function and cytochrome oxidase activity

Developing in both hypoxic or hyperoxic conditions had little effect on the respiratory function of fetal and neonatal cardiac mitochondria in the chicken. Mitochondria from embryos and neonates from all treatments had similar oxygen fluxes during LEAK and OXPHOS conditions (Fig. 3). This may be a consistent response of embryonic cardiac mitochondria to developing under hypoxic conditions in both archosaurs and mammals. As with the chicken, cardiac mitochondria oxygen flux through Complex I in a rabbit intrauterine growth restriction model did not differ from normoxic mitochondria (Guitart-Mampel et al., 2018). Cardiac mitochondria LEAK and OXPHOS oxygen flux in American alligator embryos incubated at 10% oxygen did not differ from the normoxic controls (Galli et al., 2016). It may be that OXPHOS of cardiac mitochondria from the archosaur linage are resistant to altered developmental oxygen levels. Although no other studies have examined the development of mitochondrial function in response to hyperoxic conditions, it has been shown that hypoxic incubation compromises stroke volume and cardiac output in developing chicken embryos (Jonker et al., 2015).

A number of studies have found that hypoxic fetal development influences cardiac mitochondria cytochrome oxidase activity. Fetal guinea pig hearts, in response to hypoxic fetal exposure had lower activity levels of cytochrome oxidase activities (Al-Hasan et al., 2013). This lower cytochrome oxidase activity was maintained in 90-day old guinea pig hearts from those developing under hypoxia. The only effect of hypoxia on mitochondria function observed in the current stuidy were small alterations in the cytochrome oxidase oxygen flux. Hypoxic hearts tended to have inconsistently lower respiration rates through cytochrome oxidase than normoxic or hyperoxic hearts.

A number of studies have examined the effect of fetal developmental oxygen on cardiac mitochondria function with longer lasting effects, but only looking at the juvenile or adult stages. In a mammalian model, the guinea pig, prenatal hypoxia differentially influenced cytochrome oxidase activity in males and females when measured 90 days post birth (Al-Hasan et al., 2014; Thompson et al., 2018). Hypoxic males had a lower cytochrome oxidase activity than normoxic males, while female activity was unaltered. (Thompson et al., 2018). Similar sex-based differences were seen in adult mice that had developed under hypoxic conditions (Hellgren et al., 2021). Cardiomyocytes from juvenile snapping turtles incubated in hypoxia exhibited lower LEAK and OXPHOS mitochondrial oxygen fluxes than their normoxic counterparts (Galli et al., 2021). The changes observed in some species in the juvenile or adult in response to development under hypoxic conditions may be due to differential HIF-1 or Hand1 signaling involved in cardiac mitochondria maturation that comes into play during the transition from fetal to postnatal life when arterial oxygen levels become elevated (Breckenridge et al., 2013; Nau et al., 2002; Neary et al., 2014). Thus, differences may not appear until after the mitochondria mature after birth or hatching.

While fetal cardiac mitochondria have limited responses to hypoxic development, the mitochondria function of adult hearts exposed to hypoxia show much more alteration in their function. After hypoxic acclimation for 14 days at 11% O_2_, cardiac mitochondria in adult male rats were found to be depressed (Heather et al., 2012). OXPHOS oxygen flux and maximal ADP respiration rates were significantly lower in mitochondria from hypoxic acclimated rats than normoxic rats. In response to 14 days of 11% O_2_ exposure, cardiac muscle mitochondria from adult rats exhibited lower OXPHOS and cytochrome oxidase supported oxygen fluxes (Heather et al., 2012). These differences in the response between fetal and adult cardiac mitochondria in response to chronic hypoxia may be due to fetal cardiac cells being more resistant to hypoxia and relying more on glucose and glycolysis (Lopaschuk et al., 1991; Lopaschuk and Jaswal, 2010) or to evolved speices differences. At birth in lamb half of ATP production for cardiac cells comes from glycolysis with a switch to β-oxidation (Garbern and Lee, 2021; Lopaschuk et al., 1991; Lopaschuk and Jaswal, 2010; Neary et al., 2014).

### 4.3 Mitochondria reactive oxygen species production

There were no differences in ROS production from cardiac mitochondria from animals developing under the three different oxygen treatments. Mitochondria produce reactive oxygen species (ROS) such as H_2_O_2_ and superoxide at respiratory complexes I, II, and III (Brand, 2016; Murphy, 2009). The response of mitochondrial H_2_O_2_ production in response to acclimation under hypoxic and hyperoxic conditions has been found to increase or decrease depending on the organism, age, and method of mitochondria preparation (Du et al., 2016; Hellgren et al., 2021; Hickey et al., 2012; Treberg et al., 2018). There is limited data on the direct effects of chronic hypoxic or hyperoxic incubation on mitochondrial ROS production in fetal or neonatal cardiomyocytes of mammals or birds. Developing under chronic hypoxia or hyperoxia had no effect on the rate of reactive oxygen species production in fetal or neonatal cardiac mitochondria under leak or oxidative phosphorylation conditions used in this study (Fig. 4).

A number of studies have examined the response of juvenile or adult cardiac mitochondria from mammals and reptiles that have developed under hypoxia *in utero* or in the egg. Cardiac mitochondria from 8-month-old snapping turtles incubated under hypoxic conditions produced less H_2_O_2_ than normoxic controls under LEAK state through Complexes I and II and during reverse electron transport in the presence of succinate (Galli et al., 2021). The response in adult mice that developed *in utero* under hypoxia were dependent upon the sex of the animal (Hellgren et al., 2021). Male cardiac mitochondria produced higher levels of ROS while female cardiac mitochondria produced less. Whether similar differences in the adult cardiomyocyte mitochondrial ROS production occurs in chickens incubated in hypoxia or hyperoxia remains unknown.

While there are no differences in the ROS production under the mitochondria substrate and inhibitor conditions used in this study, it is still possible that ROS levels differ within the cardiac tissues *in vivo* under the *in ovo* incubation conditions (Murphy, 2009). The ROS production measured from permeabilized fibers must be done under high oxygen conditions in the respirometer so as oxygen flux is not limited (Li Puma et al., 2020) and thus may provide an upper estimation of ROS production capacity under the metabolic states studied. In the *in vivo* state, cardiac mitochondria will likely be in an OXPHOS state somewhere below the maximal OXPHOS state measured here and not a LEAK state, so ROS production would be lower (Murphy, 2009). Additionally, it is unclear what cellular antioxidant capacity the permeabilized cardiac fibers are capable of in our preparation. It is possible the ROS production levels are similar because of differential antioxidant capacities retained within the permeabilized cells and they were all measured at elevated oxygen levels in the respirometry chamber (Li Puma et al., 2020).

### 4.4 Conclusion

Altering developmental oxygen influenced the gross morphology of the developing chicken heart but had limited effect on the mitochondrial oxygen flux capacity or reactive oxygen species production in this study. This reinforces the idea that fetal cardiac cells are adapted to hypoxic conditions in the egg, but are also unaltered by hyperoxic incubation. Cardiomyocyte mitochondria function may be resistant to chronic alterations in oxygen availability during *in ovo* development. While there are only small differences in mitochondria function from fetal and newly hatched neonatal hearts incubated in hypoxia and hyperoxia, it is still unknown if there are any long-term effects on mitochondria function that may manifest in avian adults in response to developing in altered oxygen levels as seen in mice and turtles (Galli et al., 2021; Hellgren et al., 2021).

## CRediT authorship contribution statement

**Vanessa J. Starr:** Conceptualization, Methodology, Investigation, Formal analysis, Writing – review & editing. **Edward M. Dzialowski:** Conceptualization, Methodology, Formal analysis, Writing – original draft, Writing – review & editing.

## Declaration of competing interest

The authors declare that they have no known competing financial interests or personal relationships that could have appeared to influence the work reported in this paper.
